# Iliac vein stenting with or without ovarian vein embolization for May-Thurner syndrome-associated pelvic venous disorder in women: A received-treatment retrospective cohort study

**DOI:** 10.1016/j.jvsv.2026.102641

**Published:** 2026-10-14

**Authors:** Tianhong Cai, Rong Zhang, Kai Chen, Jianfeng He, Dianxi Chen, Tenghui Zhan

**Affiliations:** a Fujian Maternity and Child Health Hospital, Fuzhou, China; b College of Clinical Medicine for Obstetrics and Gynecology and Pediatrics, Fujian Medical University, Fuzhou, China

**Keywords:** May-Thurner syndrome, Pelvic venous disorder, Iliac vein stenting, Ovarian vein embolization, Chronic pelvic pain

## Abstract

**Objective:**

To compare clinical outcomes after iliac vein stenting (IVS) alone, simultaneous IVS plus ovarian vein embolization (OVE), and staged IVS followed by OVE in women with May-Thurner syndrome-associated pelvic venous disorder.

**Methods:**

This single-center retrospective cohort study enrolled 184 women (aged 18-65 years) with imaging-confirmed May-Thurner syndrome and hemodynamically significant pelvic venous disorder at the Fujian Maternity and Child Health Hospital (January 2021-May 2025). The primary estimand was treatment received: IVS alone without subsequent OVE (group A, n = 71), simultaneous IVS plus OVE (group B, n = 54), or staged IVS then OVE (group C, n = 59, including 45 planned staged cases and 14 staged-after-IVS reassessment cases). The primary outcome was ≥ 50% reduction in Visual Analog Scale pain score at 12 months. Confounding was addressed using multivariable regression, propensity score matching, stabilized inverse probability of treatment weighting, overlap weighting, augmented inverse probability weighting, and repeated-measures models. OVE timing analyses were exploratory. Exact-date and time-updated OVE sensitivity analyses were performed, and available follow-up was extended through August 31, 2026.

**Results:**

At 12 months, response rates were 54.9% for IVS alone, 74.1% for simultaneous IVS plus OVE, and 69.5% for staged IVS plus OVE (overall *P* = .059). Receipt of OVE was associated with higher adjusted odds of response compared with IVS alone [adjusted odds ratio (OR), 2.64; 95% confidence interval (CI), 1.21-5.73; *P* = .014], supported by inverse probability of treatment weighting (OR, 3.12; bootstrap 95% CI, 1.09-6.87). Propensity score matching (OR, 2.60; 95% CI, 0.93-7.29), overlap-weighted (OR, 2.42; 95% CI, 0.66-8.84), and augmented inverse probability weighting estimates were directionally consistent but imprecise. Planned staged and staged-after-IVS reassessment cases had 12-month response rates of 73.3% and 57.1%, respectively. Recorded 30-day events were mostly grade I; one simultaneous IVS plus OVE patient had grade IIIA coil migration requiring snare retrieval, with no sequelae. Median follow-up was 37.3 months (interquartile range, 23.7-54.2; maximum, 66.5), with 96.6% stent patency among 29 patients assessed at 60 months.

**Conclusions:**

In this received-treatment analysis, adjunctive OVE was associated with higher 12-month symptom-response rates compared with IVS alone. No statistically significant difference was observed between simultaneous and staged OVE, but this exploratory timing comparison should not be interpreted as equivalence. Because residual confounding remains and OVE adds procedure-specific events, these findings support selective consideration of OVE for patients with substantial ovarian vein reflux rather than routine incorporation for all patients.

**Article Highlights**

• **Type of Research:** Single-center retrospective received-treatment cohort study
• **Key Findings:** Among 184 women with May-Thurner syndrome-associated pelvic venous disorder, receipt of adjunctive ovarian vein embolization (OVE) was associated with higher adjusted odds of 12-month Visual Analog Scale pain response compared with iliac vein stenting alone. No statistically significant difference was observed between simultaneous and staged OVE, but this exploratory timing comparison was not designed to establish equivalence.
• **Take Home Message:** Adjunctive OVE may be considered selectively for women with May-Thurner syndrome-associated pelvic venous disorder and substantial ovarian vein reflux, but residual confounding, staged after iliac vein stenting treatment, and additional OVE-specific procedural events argue against routine combined treatment for all patients.

Pelvic venous disorder (PeVD) is a recognized cause of chronic pelvic pain (CPP) and may arise from ovarian vein reflux, iliac venous obstruction, or combined pathophysiology.^1^, ^2^ May-Thurner syndrome (MTS), anatomical compression of the left common iliac vein, is common in symptomatic iliofemoral venous disease and is frequently under-recognized in women with pelvic venous symptoms.^3^, ^4^, ^5^

Comparative data describing iliac vein stenting (IVS) alone, receipt of adjunctive ovarian vein embolization (OVE), and OVE timing remain scarce.^6^, ^7^, ^8^, ^9^, ^10^ Existing studies suggest that IVS and OVE treat different components of MTS-associated PeVD, but have not clearly defined which patients benefit from adding reflux-directed therapy or whether OVE should be performed simultaneously or after an IVS-first interval. This question is clinically relevant because many patients improve after iliac decompression alone, whereas OVE adds procedure time, cost, and embolization-specific risks.

We hypothesized that receipt of adjunctive OVE would be associated with greater 12-month symptom response than IVS alone in women with MTS-associated PeVD. We therefore compared IVS alone with received adjunctive OVE, with exploratory analyses of simultaneous vs staged OVE timing; the study was not powered to determine optimal timing. We also examined actual OVE timing and available follow-up beyond 1 year.

## Methods

### Study population

This single-center retrospective cohort study enrolled consecutive women (aged 18-65 years) who underwent endovascular treatment for MTS complicated by PeVD at the Fujian Maternity and Child Health Hospital (January 2021-May 2025). MTS was diagnosed by intravascular ultrasound (IVUS) or computed tomography venography (CTV) demonstrating >50% left common iliac vein luminal reduction or extrinsic compression with collaterals. PeVD was confirmed when ≥2 of: CPP ≥6 months; pelvic/perineal/vulvar varicosities; dyspareunia or postcoital pain; cyclical exacerbation, with competing pathology excluded. Patients with acute iliofemoral deep vein thrombosis (DVT), previous ipsilateral endovascular intervention, coagulopathy, contraindication to anticoagulation, active malignancy, or missing 12-month primary outcome data were excluded. The primary analysis used a received-treatment framework: IVS alone without subsequent OVE (group A, n = 71), simultaneous IVS plus OVE (group B, n = 54), and staged IVS plus OVE (group C, n = 59). Group C included 45 planned staged patients with OVE and 14 patients initially treated with IVS who subsequently underwent clinically indicated OVE for persistent symptoms. Data were retrieved from standardized medical records with independent verification. Preprocedural CTV reports were reviewed for left renal vein compression, which was distinguished from clinically diagnosed Nutcracker syndrome.

### Procedures

All endovascular procedures were performed by one of three senior vascular surgeons, each with >50 previous IVS procedures. Protocols were standardized across operators and remained unchanged during enrolment, although operator-specific preferences for staged OVE timing may have introduced clustering not fully captured in the primary analysis.

For IVS, ipsilateral femoral access was obtained under ultrasound guidance. IVUS confirmed stenosis site and severity, guided stent sizing, and verified procedural success, defined as residual stenosis <30% on completion venography and IVUS.

For OVE, right internal jugular or femoral access was used. Selective venography confirmed reflux, and embolization used platinum microcoils plus 3% sodium tetradecyl sulfate foam until reflux ceased. Selective ovarian venography was performed with the microcatheter within 1 cm of the ostium at 2-3 mL/s (6-10 mL total contrast); reflux was graded mild (retrograde column ≤ 5 cm), moderate (>5-15 cm), or severe (>15 cm).

In group B, IVS and OVE were performed in the same session, with IVS completed first to reassess pelvic hemodynamics. In group C, IVS was followed by OVE after interval reassessment within a staged-treatment pathway. Treatment timing reflected anatomy, reflux burden, symptoms, physician judgment, and patient preference rather than random assignment. Simultaneous OVE was generally considered for marked baseline ovarian reflux with pelvic pooling, whereas staged OVE was used when reassessment after outflow restoration was clinically preferred.

OVE was selected when ultrasound study/CTV and venography showed a clear ovarian-vein escape point with substantial reflux or pelvic pooling judged concordant with symptoms and the patient agreed. IVS alone was used when outflow obstruction was considered dominant. Planned staging was chosen when a shorter initial procedure, lower immediate cost, or interval reassessment was preferred; 14 patients received OVE later for persistent symptoms after IVS.

### Variables

The exposure variable was treatment received. Baseline was defined immediately before index IVS, and follow-up time was anchored to the index IVS date for all groups; staged OVE occurred within this follow-up interval and did not reset the 12-month clock. Fourteen patients initially managed with IVS alone subsequently underwent OVE for persistent symptoms and were analyzed in the staged OVE group for the primary received-treatment estimand. The primary outcome was ≥ 50% reduction in 0 to 10 cm Visual Analog Scale (VAS) pain^11^ from baseline to 12 months, consistent with Initiative on Methods, Measurement, and Pain Assessment in Clinical Trials recommendations.^12^ Blinded research coordinators assessed VAS at baseline and 1, 3, 6, and 12 months. Secondary outcomes were Venous Insufficiency Epidemiological and Economic Study-Quality of Life (VEINES-QOL),^13^, ^14^ 36-Item Short Form Health Survey (SF-36) Physical Component Summary (PCS)/ Mental Component Summary (MCS),^15^, ^16^ 12-month primary stent patency, 30-day complications, and 12-month reintervention. All patients received rivaroxaban 10 mg once daily for 3 months. Available follow-up was extended through August 31, 2026; 24-, 36-, 48-, and 60-month analyses included patients who had reached each time point.

Patient-level Symptoms-Varices-Pathophysiology (SVP) classification was assigned retrospectively from recorded baseline symptom domains, variceal manifestations, and pathophysiologic components. The S domain reflected the number and severity of pelvic venous symptoms; the V domain reflected recorded variceal manifestations and pelvic venous pooling; and the P domain incorporated iliac venous obstruction, left renal/branch reflux patterns when recorded, and pelvic reflux components. SVP classification was used descriptively to characterize baseline phenotype and treatment selection rather than as a treatment-effect modifier. Left renal vein compression and clinical Nutcracker syndrome were recorded separately.

### Statistical analysis

Covariates were selected a priori: age, body mass index, parity, symptom duration, IVUS stenosis severity, ovarian vein diameter, pelvic pooling grade, baseline VAS, and previous DVT. Baseline and 12-month primary outcome data were complete; longitudinal models used available repeated observations under a missing-at-random assumption. No formal sample-size calculation was performed. All secondary end point, subgroup, and OVE-timing analyses were exploratory; no formal multiplicity correction was applied to secondary end point *P* values. Sensitivity analyses used overlap weighting and augmented inverse probability weighting (AIPW) with baseline covariates available for all 184 patients. Because 14 patients received staged-after-IVS OVE after initial IVS, timing was also examined by separating planned staged OVE from staged-after-IVS reassessment OVE and by describing 3-month landmark exposure status.

Continuous variables are summarized as median [interquartile range (IQR)] or mean [standard deviation (SD)], and categorical variables as n (%). Three-group comparisons used Kruskal-Wallis tests for median-reported variables, one-way analysis of variance for mean-reported variables, and Pearson *X*^*2*^ tests for categorical variables. Tests were two-sided. Standardized mean difference (SMD) denominators used the unadjusted pooled within-group SD; matched baseline characteristics were assessed using SMD rather than significance tests.

Treatment selection followed an allocation-by-indication structure: patients with greater reflux burden and more complex SVP phenotypes were more likely to receive OVE. The primary comparison was received OVE (groups B + C) vs IVS alone, analyzed with multivariable logistic regression adjusted for age, body mass index, parity, symptom duration, IVUS stenosis severity, ovarian vein diameter, baseline VAS, and previous DVT. Propensity score matching (PSM) and stabilized inverse probability of treatment weighting (IPTW) used propensity scores estimated from age, parity, symptom duration, IVUS stenosis, ovarian vein diameter, pelvic pooling grade, baseline VAS, and previous DVT. Matching was 1:1 without replacement using a 0.2-SD logit caliper; the matched odds ratio (OR) accounted for pairs. IPTW weights were truncated at the 99th percentile, and percentile confidence intervals (CIs) used 2000 patient-level bootstrap resamples with propensity-score refitting. Supplementary Table I, *A* and *B* (online only) reports balance and matched characteristics. The expanded propensity model for overlap weighting included baseline demographics, symptom burden, IVUS stenosis severity, ovarian vein diameter, pelvic pooling grade, baseline VAS, baseline quality-of-life scores, previous DVT, dyspareunia, urinary symptoms, and lower-limb varicosities. Pelvic pooling and SVP were treated mainly as treatment-selection context. To address treatment timing, an exact-date 12-month sensitivity analysis counted OVE only when received before the assessment, and repeated VAS was analyzed with OVE as a time-updated covariate in a population-averaged Gaussian model. Extended follow-up was descriptive and used available cases without imputation. Analyses used Python 3.13.14, statsmodels 0.14.6, and scikit-learn 1.9.0.

## Ethics statement

This study was approved by the Institutional Review Board of the Fujian Maternity and Child Health Hospital (Approval No. 2026KY218-01) and was conducted in accordance with the Declaration of Helsinki and applicable national regulations. Given the retrospective nature of the study and the use of fully anonymized patient data extracted from existing medical records, the requirement for individual informed consent was waived by the ethics committee. All patient identifiers were removed before data analysis, and data were stored in an encrypted, access-controlled database accessible solely to authorized study personnel. No study-specific procedures were performed.

As a fully retrospective analysis of existing clinical data, this study was not prospectively registered in a clinical trials registry. End point definitions, covariate selection, and sensitivity analyses were documented before final database lock; all subgroup analyses are presented as exploratory.

## Results

Between January 2021 and May 2025, a total of 184 women were included in the received-treatment cohort: IVS alone without subsequent OVE (group A, n = 71), simultaneous IVS plus OVE (group B, n = 54), and staged IVS plus OVE (group C, n = 59). Group C included 59 staged OVE cases, including 45 prespecified staged cases and 14 staged-after-IVS reassessment cases for persistent symptoms. Baseline characteristics are reported in Table I; parity differed across groups (*P* = .011). Preprocedural CTV identified left renal vein compression in 23 patients (12.5%), but none had clinically diagnosed Nutcracker syndrome, hematuria, or proteinuria.

**Table I:** Baseline characteristics of the received-treatment cohort

Characteristics	Group A: IVS alone (N = 71)	Group B: simultaneous IVS + OVE (N = 54)	Group C: staged IVS + OVE (N = 59)	*P* value
Demographics				
Age, years, median (IQR)	36.0 (30.0-43.0)	32.0 (24.0-40.0)	38.0 (31.0-47.5)	.061
BMI, kg/m^2^, mean ± SD	23.8 (3.3)	22.9 (3.1)	23.1 (3.4)	.275
Parity, median (IQR)	2.0 (1.0-2.0)	2.0 (1.0-3.0)	2.0 (1.0-3.0)	.011
Symptom duration, months, median (IQR)	21.0 (10.1-53.0)	45.0 (23.7-68.2)	24.0 (17.5-44.5)	.004
Clinical presentation				
Chronic pelvic pain	71 (100.0)	54 (100.0)	59 (100.0)	—
Dyspareunia	40 (56.3)	40 (74.1)	40 (67.8)	.105
Dysuria/urgency	20 (28.2)	24 (44.4)	25 (42.4)	.114
Lower limb varicosities	26 (36.6)	17 (31.5)	20 (33.9)	.834
Previous DVT history	11 (15.5)	7 (13.0)	9 (15.3)	.914
Imaging and hemodynamic parameters				
Iliac vein stenosis by IVUS, %, median (IQR)	66.0 (57.5-77.5)	69.0 (59.5-82.4)	66.0 (56.4-78.4)	.439
Iliac vein stenosis > 70%	33 (46.5)	23 (42.6)	27 (45.8)	.904
Ovarian vein diameter, mm, median (IQR)	6.6 (5.5-8.2)	10.6 (9.2-13.2)	9.7 (7.3-11.7)	<.001
Ovarian vein diameter > 6 mm	38 (53.5)	46 (85.2)	50 (84.7)	<.001
Ovarian vein diameter > 8 mm	21 (29.6)	45 (83.3)	39 (66.1)	<.001
Pelvic venous pool grade ≥ 2	21 (29.6)	50 (92.6)	46 (78.0)	<.001
Baseline symptom scores				
VAS pain score, median (IQR)	6.0 (4.9-7.3)	6.9 (5.6-8.0)	7.2 (6.0-9.0)	.008
VEINES-QOL, mean ± SD	48.8 (9.0)	45.4 (9.3)	45.7 (10.1)	.077
SF-36 PCS, mean ± SD	40.8 (9.3)	39.8 (8.5)	42.0 (8.3)	.407
SF-36 MCS, mean ± SD	43.7 (9.2)	42.8 (9.1)	44.5 (9.7)	.625
Procedural details				
Dedicated venous stent	58 (81.7)	46 (85.2)	51 (86.4)	.741
Bilateral stenting	7 (9.9)	5 (9.3)	5 (8.5)	.964
Bilateral OVE	—	37 (68.5)	29 (49.2)	—
Interval IVS to OVE, months, median (IQR)	—	—	5.3 (4.1-6.1)	—
Left renal vein compression on CTV	5 (7.0)	7 (13.0)	11 (18.6)	.137

*ANOVA*, Analysis of variance; *BMI*, body mass index; *CTV*, computed tomography venography; *DVT*, deep vein thrombosis; *IQR*, interquartile range; *IVS*, iliac vein stenting; *IVUS*, intravascular ultrasound; *MCS*, Mental Component Summary; *OVE*, ovarian vein embolization; *PCS*, Physical Component Summary; *SD*, standard deviation; *SF-36*, 36-Item Short Form Health Survey; *VAS*, Visual Analog Scale; *VEINES-QOL*, Venous Insufficiency Epidemiological and Economic Study-Quality of Life.

Continuous-variable summaries use the 184-patient analytic dataset. *P* values are from Kruskal-Wallis tests for median-reported variables, one-way ANOVA for mean-reported variables, and Pearson *X*^*2*^ tests for categorical variables.

Categorical data are shown as number (%).

Symptom duration differed among groups (*P* = .004), with the simultaneous IVS plus OVE group reporting the longest median duration of pelvic symptoms (45 months; IQR, 23.7-68.2) compared with IVS alone (21 months; IQR, 10.1-53.0) and staged IVS plus OVE (24 months; IQR, 17.5-44.5). CPP was universal, whereas dyspareunia, urinary symptoms, lower-limb varicosities, and previous DVT were not significantly different across groups.

Ovarian vein reflux burden remained greater in the OVE-treated groups than IVS-only patients. Median ovarian vein diameter was 6.6 mm (IQR, 5.5-8.2) in group A, 10.6 mm (IQR, 9.2-13.2) in group B, and 9.7 mm (IQR, 7.3-11.7) in group C (*P* < .001). Pelvic venous pooling grade ≥2 was present in 29.6%, 92.6%, and 78.0% of patients, respectively (*P* < .001). Iliac vein stenosis severity was comparable across groups. Among 113 OVE-treated patients, selective venography graded reflux as mild in 17, moderate in 36, and severe in 60.

SVP phenotypes also differed across received-treatment groups (Supplementary Table II, online only). More severe symptom-domain classification (S3) was observed in 49.3% of IVS-alone patients, 64.8% of simultaneous IVS plus OVE patients, and 71.2% of staged IVS plus OVE patients (*P* = .030). Variceal- and pathophysiology-domain distributions differed more strongly across groups (*P* < .001 and *P* < .001, respectively), with OVE-treated patients more commonly showing combined obstructive and reflux phenotypes. These SVP differences support the presence of allocation by phenotype and were interpreted as treatment-selection context rather than as evidence of causal subgroup effects.

Baseline VAS scores differed across treatment categories. Median VAS pain score was 6.0 (IQR, 4.9-7.3) in group A, 6.9 (IQR, 5.6-8.0) in group B, and 7.2 (IQR, 6.0-9.0) in group C (*P* = .008). Baseline VEINES-QOL, SF-36 PCS, and SF-36 MCS scores did not differ significantly across groups.

Procedural characteristics were generally uniform. Dedicated venous stents were used in 81.7%, 85.2%, and 86.4% of groups A, B, and C, respectively (*P* = .741), and bilateral stenting rates were similar. Among OVE-treated patients, bilateral OVE was performed in 68.5% of simultaneous cases and 49.2% of staged cases; the median interval from IVS to OVE in the staged group was 5.3 months (IQR, 4.1-6.1).

PSM yielded 34 IVS-alone and 34 received-OVE patients; the estimate was directionally consistent but imprecise (paired OR, 2.60; 95% CI, 0.93-7.29; *P* = .069; Supplementary Table I, *B*, online only and Table II). Stabilized IPTW was concordant (OR, 3.12; bootstrap 95% CI, 1.09-6.87; Supplementary Table III, *A*, online only). Maximum absolute SMD was 0.65 after IPTW and 0.30 after PSM, indicating residual imbalance (Supplementary Table I, *A*, online only). In the expanded propensity model, overlap weighting improved maximum absolute SMD from 1.33 before weighting to 0.12 after weighting (Supplementary Table III, *B*, online only). Overlap-weighted response rates were 68.7% for received OVE and 47.5% for IVS alone (risk difference, 21.2-percentage points; bootstrap 95% CI, −2.7 to 39.4); the marginal OR was 2.42 (95% CI, 0.66-8.84; *P* = .181). AIPW estimated a directionally similar 19.1 percentage-point response difference (bootstrap 95% CI, −9.5 to 39.4).

**Table II:** Primary outcome: proportion of patients achieving ≥ 50% reduction in pelvic pain Visual Analog Scale (*VAS*) score at 12 months (received-treatment analysis)

Analysis	IVS alone (N = 71)	Simultaneous IVS + OVE (N = 54)	Staged IVS + OVE (N = 59)	Effect estimate (95% CI)	*P* value
Responders	39 (54.9)	40 (74.1)	41 (69.5)	Overall, three-group comparison	.059
Adjusted logistic regression (reference: IVS alone)	Reference	3.06 (1.20-7.78)	2.36 (1.00-5.58)	Group-specific aORs vs IVS alone	.019; .050
Any received OVE vs IVS alone, aOR (95% CI)	Reference	Included in received-OVE group	Included in received-OVE group	2.64 (1.21-5.73)	.014
Simultaneous vs staged OVE, aOR (95% CI)	Not applicable	Comparator	Reference	1.31 (0.52-3.29)	.566
PSM sensitivity: received OVE vs IVS alone, 34 matched pairs	Reference	Included in received-OVE group	Included in received-OVE group	2.60 (0.93-7.29)	.069
Stabilized IPTW sensitivity: received OVE vs IVS alone	Reference	Included in received-OVE group	Included in received-OVE group	3.12 (1.09-6.87)	—
OVE received by actual 12-month assessment date, aOR (95% CI)	Reference	Included if OVE received by assessment	Included if OVE received by assessment	2.44 (1.13-5.25)	.023
Time-updated OVE exposure in repeated VAS model, coefficient (95% CI)	Reference pathway	Exposure active from OVE date	Exposure active from OVE date	−0.63 (−0.91 to −0.36)	<.001

*aOR*, Adjusted odds ratio; *AR*, autoregressive; *CI*, confidence interval; *GEE*, generalized estimating equations; *IPTW*, inverse probability of treatment weighting; *IVS*, iliac vein stenting; *OVE*, ovarian vein embolization; *PSM*, propensity score matching; *VAS*, visual analog scale.

Categorical data are shown as number (%).

The primary estimand was treatment received. The exact-date analysis classified one planned staged patient whose OVE occurred after the 12-month assessment as unexposed at that assessment. The time-updated coefficient is the adjusted mean VAS difference, whereas OVE exposure was active in a Gaussian GEE with AR (1) working correlation. All timing analyses are exploratory and do not control time-varying confounding by indication.

PSM estimates use matched-pair discordance. The IPTW interval is a percentile interval from 2000 patient-level bootstrap resamples; no separate *P* value is reported.

At 12 months, ≥ 50% VAS pain reduction was achieved by 54.9% (39/71), 74.1% (40/54), and 69.5% (41/59) of groups A, B, and C, respectively (overall *P* = .059; Table II; Fig 1). In adjusted models, simultaneous IVS plus OVE [adjusted OR (aOR), 3.06; 95% CI, 1.20-7.78; *P* = .019] and staged IVS plus OVE (aOR, 2.36; 95% CI, 1.00-5.58; *P* = .050) were directionally favorable vs IVS alone; pooled received OVE was independently associated with response (aOR, 2.64; 95% CI, 1.21-5.73; *P* = .014).

**Fig 1:**
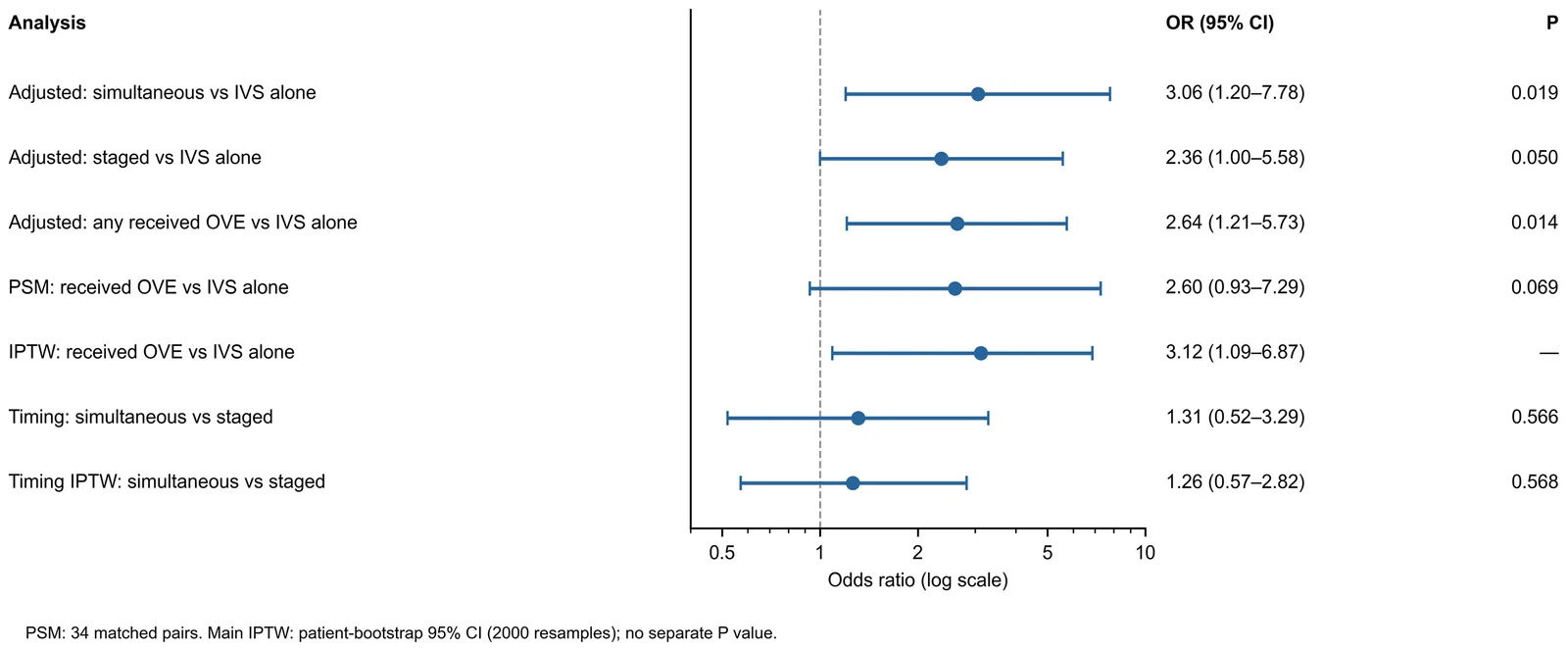
Primary outcome: at least 50% reduction in Visual Analog Scale (VAS) pain score at 12 months. Forest plot of odds ratios (*OR*) and 95% confidence intervals (*CIs*) from adjusted logistic regression, propensity score matching (34 pairs), stabilized inverse probability of treatment weighting (*IPTW*), and exploratory ovarian vein embolization (*OVE*) timing analyses. The matched estimate accounts for pairs. The received-OVE IPTW confidence interval uses 2000 patient-level bootstrap resamples with propensity-score refitting; no separate *P* value is reported for this estimate. Odds ratios are plotted on a log scale. Values above one favor the OVE group for comparisons against iliac vein stenting (*IVS*) alone, and simultaneous OVE for comparisons against staged OVE. *PSM*, Propensity score matching.

In continuous-outcome analysis, received OVE was associated with an additional 0.88-point 12-month VAS reduction after adjustment (95% CI, 0.13-1.62; *P* = .022).

PSM, overlap-weighting, and AIPW estimates were directionally consistent but imprecise, reflecting the reduced effective comparison set after accounting for severe allocation by reflux burden. Among OVE-treated patients, simultaneous and staged OVE did not differ significantly after adjustment (aOR, 1.31; 95% CI, 0.52-3.29; *P* = .566; Table II and Supplementary Table III, *A*, online only), an underpowered comparison that should not be read as equivalence.

Within group C, 45 patients had planned staged OVE at baseline or index IVS and 14 had staged-after-IVS reassessment OVE for persistent symptoms. Twelve-month response rates were 73.3% (33/45) and 57.1% (8/14), respectively, (Fisher exact *P* = .322; Supplementary Table III, *C*, online only). Mean IVS-to-OVE intervals were 174.4 and 157.3 days, respectively. At the 3-month landmark, all simultaneous cases had already received OVE, whereas all staged cases had not yet received OVE; therefore, timing analyses were interpreted as early/simultaneous vs deferred/staged pathway descriptions rather than causal timing effects.

Exact dates showed that one planned staged OVE occurred after the 12-month assessment. Classifying OVE by receipt before the actual assessment produced an aOR of 2.44 (95% CI, 1.13-5.25; *P* = .023). In a time-updated repeated-VAS model, current OVE exposure was associated with a 0.63-point lower VAS score (95% CI, 0.36-0.91 lower; *P* < .001). This analysis remained descriptive because treatment timing was nonrandom and time-varying confounding could not be fully controlled.

To explore whether OVE timing was associated with pain outcomes, Groups B and C were compared in adjusted and weighted analyses. Weighted response rates were 71.7% and 66.7% for simultaneous and staged OVE, respectively, without a statistically significant difference (weighted OR, 1.26; 95% CI, 0.57-2.82; *P* = .568). The study was not powered to determine the optimal timing of OVE, and these findings should not be interpreted as demonstrating equivalence between timing strategies.

Median follow-up was 37.3 months (IQR, 23.7-54.2), with a maximum of 66.5 months. Among patients due for assessment, data were available for 140/153 (91.5%), 98/105 (93.3%), 74/80 (92.5%), and 29/35 (82.9%) at 24, 36, 48, and 60 months, respectively; corresponding clinical response rates were 60.7%, 60.2%, 64.9%, and 55.2%, and stent patency was 99.3%, 99.0%, 98.6%, and 96.6% (Supplementary Table III, *D*, online only and Supplementary Table III, *E*, online only).

VAS pain scores decreased progressively across all groups (Supplementary Table III, *F*, online only; Fig 2). Group C exhibited a higher score at 1 month (5.0 vs 4.0 in groups A and B; *P* < .001), reflecting the pre-OVE interval for staged treatment. From 3 months onward, VAS scores converged across groups.

**Fig 2:**
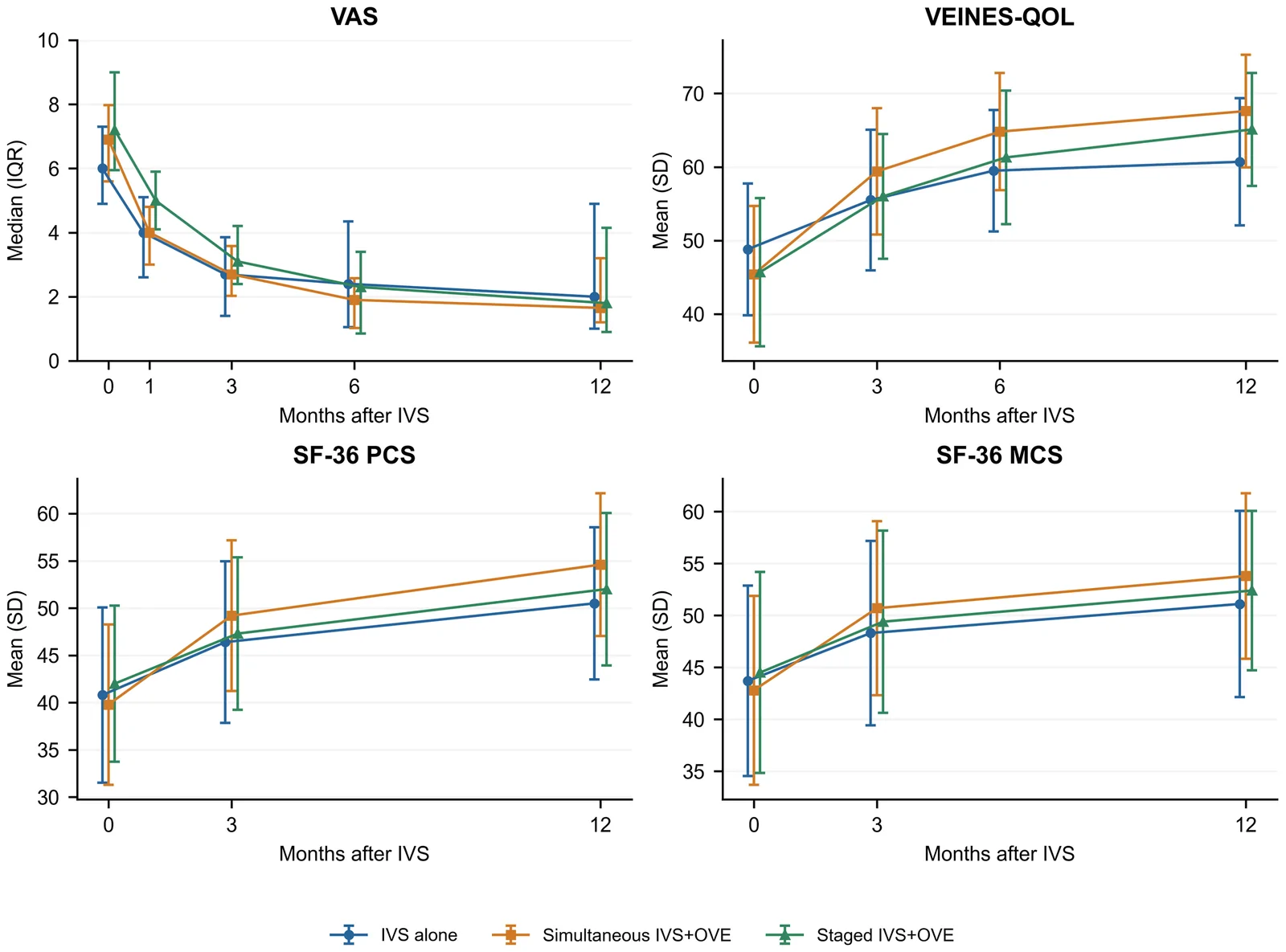
Symptoms and quality of life from baseline through 12 months. Panels show Visual Analog Scale (*VAS*) pain score **(A)**, Venous Insufficiency Epidemiological and Economic Study-Quality of Life (*VEINES-QOL*; **B)**, 36-Item Short Form Health Survey (*SF-36*) physical component summary (*PCS*; **C**), and SF-36 mental component summary (*MCS*; **D)** for iliac vein stenting (*IVS*) alone (n = 71), simultaneous IVS plus ovarian vein embolization (*OVE*; n = 54), and staged IVS plus OVE (n = 59). Points and error bars represent median and interquartile range for VAS, and mean and sample standard deviation for quality-of-life scores. *IQR*, Interquartile range.

VEINES-QOL improved in all groups. At 12 months, mean VEINES-QOL was 60.7 (SD, 8.7) in group A, 67.6 (SD, 7.7) in group B, and 65.1 (SD, 7.7) in group C (*P* < .001).

Secondary end points and subgroup analyses were exploratory. Reported *P* values were unadjusted, and no interaction test identified a statistically significant treatment-effect difference between subgroups.

SF-36 PCS improved across groups, with a 12-month between-group difference (group A, 50.5; group B, 54.6; group C, 52.0; *P* = .018). SF-36 MCS improved across groups without significant between-group differences at 12 months (*P* = .199).

Primary stent patency at 12 months was high across groups (group A, 100.0%; group B, 100.0%; group C, 98.3%; *P* = .345; Supplementary Table III, *G*, online only; Fig 3). Reintervention and symptom recurrence rates did not differ statistically across groups.

**Fig 3:**
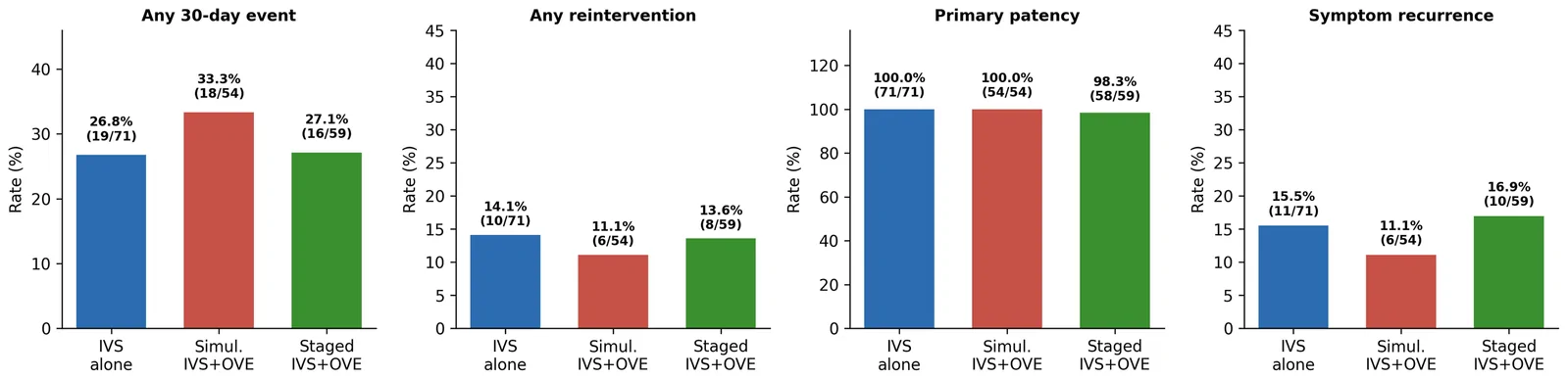
Stent patency, reintervention, recurrence, and 30-day procedural events. Bars summarize 12-month primary stent patency, in-stent restenosis or thrombosis when applicable, reintervention, symptom recurrence, and recorded 30-day events by treatment group. *IVS*, Iliac vein stenting; *OVE*, ovarian vein embolization.

Exploratory subgroup analyses showed directionally greater response with received OVE in several strata, including ovarian vein diameter >6 mm and symptom duration >2 years, but no interaction test reached statistical significance (Supplementary Table III, *H*, online only; Fig 4). These findings should be used to generate hypotheses for future prospective patient selection studies rather than define a definitive treatment threshold.

**Fig 4:**
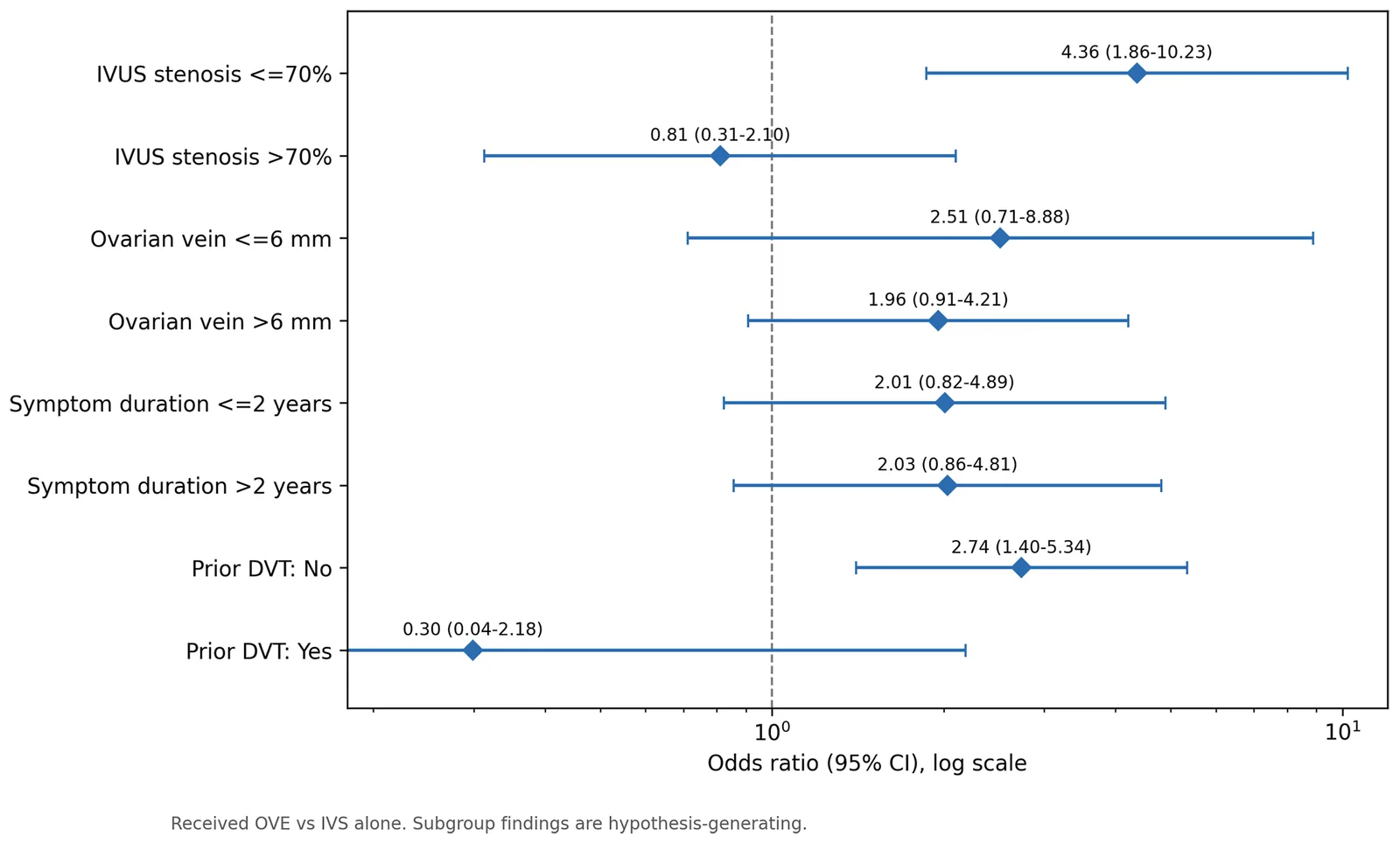
Exploratory subgroup analysis of the primary outcome. Odds ratios compare received ovarian vein embolization (*OVE*) vs iliac vein stenting (IVS) alone within baseline strata. Subgroup analyses were exploratory and should not be interpreted as validated treatment-selection thresholds. *CI*, confidence interval; *DVT*, deep vein thrombosis; *IVUS*, intravascular ultrasound.

Multivariable logistic regression identified receipt of OVE as the only independent predictor of the primary outcome in the received-treatment framework (aOR, 2.46; 95% CI, 1.08-5.61; *P* = .032; Supplementary Table III, *I*, online only). Ovarian vein diameter was not independently associated with response after accounting for treatment received (aOR, 0.99 per mm; 95% CI, 0.89-1.09; *P* = .788).

Any recorded 30-day event occurred in 26.8%, 33.3%, and 27.1% of groups A, B, and C, respectively (*P* = .682; Supplementary Table IV, online only). Most events were grade I puncture-site ecchymosis, transient chest tightness with negative computed tomography pulmonary angiography, or low-grade postembolization fever. Stent thrombosis and restenosis were recorded separately, and no procedure-related death was recorded.

The clinical decision involves a benefit-harm trade-off: received OVE was associated with an absolute 12-month response advantage of approximately 15% to 19% compared with IVS alone, while adding OVE-specific minor events, mainly low-grade postembolization fever. One simultaneous IVS plus OVE patient had coil migration from the left ovarian vein to the renal vein ostium; the coil was retrieved with a snare via internal jugular access, hospitalization was prolonged by 1 day, and no sequelae occurred. No pulmonary embolism, major bleeding, access-site complication requiring intervention, or 30-day mortality was recorded. This safety profile supports individualized selection and shared decision-making rather than routine combined intervention for all patients.

## Discussion

In this single-center retrospective cohort of 184 women with MTS-associated PeVD, receipt of adjunctive OVE was associated with higher adjusted odds of 12-month symptomatic response than IVS alone. IPTW was concordant, whereas PSM, overlap-weighting, and AIPW were directionally consistent but imprecise after accounting for severe allocation by reflux burden. No significant difference was observed between simultaneous and staged OVE; the timing analysis was exploratory and not designed to establish equivalence. Exact-date and time-updated analyses were directionally consistent, and available-case follow-up extended to 60 months; later estimates were descriptive because the risk sets became smaller.

The association between adjunctive reflux-directed treatment and symptom response is consistent with Senechal et al,^17^ who reported improved or resolved pelvic pain after transcatheter embolization. That cohort included relatively few patients with concurrent IVS for MTS and did not provide formal between-group estimates. The present study adds received-treatment estimates, propensity-based sensitivity analyses, and a procedural safety profile in patients managed for both iliac obstruction and ovarian reflux.

Our findings contrast with reports of substantial pain relief after IVS alone^18^, ^19^ and with data suggesting iliac compression does not modify outcomes after primary embolisation.^20^ Differences in end point definitions, imaging criteria, IVUS use, follow-up duration, and reflux inclusion criteria may explain these discrepancies.

Mechanistically, MTS-associated PeVD may reflect both antegrade iliac obstruction and retrograde ovarian vein reflux. Iliac decompression may reduce some reflux, but a dilated, valve-incompetent ovarian vein can maintain pelvic venous hypertension despite stent patency, providing the rationale for selective combined intervention.

The 14 staged-after-IVS reassessment cases illustrate that some patients treated with an IVS-first strategy proceed to OVE after interval reassessment. Their response rate was lower than that of planned staged cases, although the subgroup was small, and the difference was not statistically significant. Whether proactive simultaneous treatment, planned staged treatment, or IVS-first reassessment is superior cannot be determined without prospective timing rules. These pathways reflect selective practice: IVS alone was favored when obstruction appeared dominant, whereas OVE required clinically concordant reflux and patient agreement.

Strengths include a relatively large single-center MTS-associated PeVD cohort, dual-modality imaging confirmation, a validated primary pain-response end point, structured outcome assessment, standardized procedural protocols, and both matching and weighting sensitivity analyses.

This study has limitations. The retrospective, single-center design precludes causal inference, and residual confounding by central sensitization, patient preference, reflux severity, and operator-specific patterns cannot be excluded. The received-treatment framework reflects actual procedural exposure but introduces possible time-related classification bias for staged OVE, and the time-updated model did not eliminate confounding by indication. Residual imbalance persisted after matching and IPTW. Although overlap weighting improved measured balance, the overlap-weighted and AIPW estimates were imprecise and should be interpreted as sensitivity analyses within the common-support population. SVP classification was assigned retrospectively and should be interpreted descriptively rather than as a validated selection algorithm. Complication severity and management were abstracted from chart review, so minor events may be under-recorded. Although follow-up extended to 60 months, not all patients had reached later visits and the 60-month estimates were based on 29 patients; and outcomes centered on VAS pain rather than the full PeVD morbidity spectrum, including sexual function, urinary symptoms, fertility concerns, work participation, cost, and patient preference. Subgroup findings are hypothesis-generating only.

## Conclusions

In this received-treatment cohort of women with MTS-associated PeVD and varying degrees of ovarian/pelvic venous reflux burden, adjunctive OVE was associated with higher adjusted odds of 12-month symptomatic response compared with IVS alone. No statistically significant difference was observed between simultaneous and staged OVE, but this exploratory finding should not be interpreted as equivalence. Because the evidence is observational, overlap-weighted and AIPW estimates were imprecise, and OVE introduced procedure-specific events despite a low serious-complication burden, these findings support selective consideration of OVE through shared decision-making rather than routine combined treatment for all patients. Prospective multicenter studies with longer follow-up, prospectively defined staged-treatment timing, comprehensive patient-reported outcomes, and health-economic evaluation are warranted.

## Author Contributions

Conception and design: TC, TZ

Analysis and interpretation: TC, TZ

Data collection: TC, RZ, KC, JH, DC

Writing the article: TC, TZ

Critical revision of the article: TC, RZ, KC, JH, DC, TZ

Final approval of the article: TC, RZ, KC, JH, DC, TZ

Statistical analysis: TC, TZ

Obtained funding: Not applicable

Overall responsibility: TZ

## Consent for publication

Not applicable.

## Data availability

The datasets used to support the findings of this study and analyzed during the current study are available from the corresponding author upon reasonable request.

## Competing interests

The authors declare no competing interests.

## Declaration of generative AI and AI-assisted technologies in the writing process

During the preparation of this work, the authors used OpenAI Codex (models: GPT-5.5, GPT-5.6-sol, and GPT-6-astra) to improve readability and language clarity. The authors reviewed and edited all AI-assisted content and take full responsibility for the final content of the manuscript for the publication.

## Funding

This research received no specific grant from any funding agency in the public, commercial, or not-for-profit sectors.

## Disclosures

The authors declared no potential conflicts of interest with respect to the research, authorship, and publication of this article.

## Appendix (online only)

Supplementary Table I (online only)

Supplementary Table II (online only)

Supplementary Table III (online only)

Supplementary Table IV (online only)
