# Supplementary material for: Iliac vein stenting with or without ovarian vein embolization for May-Thurner syndrome-associated pelvic venous disorder in women: A received-treatment retrospective cohort study
Source: J Vasc Surg Venous Lymphat Disord. 2026 Oct 14;15(1):102641. doi: 10.1016/j.jvsv.2026.102641 (PMC13769316; doi:10.1016/j.jvsv.2026.102641)
Supplement: Supplementary file 1 [file mmc1.docx]

# Supplementary Table S1. Propensity-score balance diagnostics and matched-cohort characteristics

Absolute standardized mean differences are shown for the received OVE versus IVS-alone comparison before adjustment, after stabilized inverse probability weighting with 99th-percentile truncation, and after 1:1 propensity-score matching where available.

A. Propensity-score balance diagnostics

| **Variable** | **Before weighting** | **After IPTW** | **After PSM** |
| --- | --- | --- | --- |
| Age, years | 0.01 | 0.10 | 0.21 |
| BMI, kg/m² | 0.24 | 0.31 | 0.06 |
| Parity | 0.50 | 0.02 | 0.16 |
| Symptom duration, months | 0.07 | 0.16 | 0.08 |
| IVUS stenosis, % | 0.09 | 0.02 | 0.11 |
| Ovarian vein diameter, mm | 1.13 | 0.08 | 0.17 |
| Baseline VAS pain score | 0.41 | 0.23 | 0.14 |
| Prior DVT history | 0.04 | 0.17 | 0.00 |
| Pelvic venous pooling grade | 1.33 | 0.65 | 0.00 |
| Baseline VEINES-QOL | 0.35 | 0.02 | 0.30 |

Truncated stabilised weights ranged from 0.39 to 8.47. Effective sample sizes were 21.3 for IVS alone and 55.6 for received OVE. Maximum absolute SMD was 1.33 before adjustment, 0.65 after IPTW, and 0.30 after PSM.

OVE = ovarian vein embolisation; IVS = iliac vein stenting; IPTW = inverse probability of treatment weighting; PSM = propensity-score matching; SMD = standardized mean difference; VAS = visual analogue scale; DVT = deep vein thrombosis.

B. Baseline characteristics of the propensity score-matched cohort (34 pairs)

| **Characteristic** | **IVS alone (n=34)** | **Received OVE (n=34)** | **Absolute SMD** |
| --- | --- | --- | --- |
| Age, years | 37.32 (11.83) | 39.85 (12.79) | 0.21 |
| BMI, kg/m² | 23.16 (3.49) | 22.95 (3.59) | 0.06 |
| Parity | 1.50 (0.51) | 1.62 (1.04) | 0.16 |
| Symptom duration, months | 55.70 (59.68) | 51.78 (53.82) | 0.08 |
| IVUS stenosis, % | 68.02 (13.82) | 66.53 (13.46) | 0.11 |
| Ovarian vein diameter, mm | 7.87 (2.88) | 8.41 (2.52) | 0.17 |
| Baseline VAS pain score | 6.57 (1.77) | 6.31 (1.80) | 0.14 |
| Prior DVT history | 6 (17.6%) | 6 (17.6%) | 0.00 |
| Pelvic venous pooling grade | 1.50 (0.99) | 1.50 (0.90) | 0.00 |
| Baseline VEINES-QOL | 49.37 (8.94) | 46.57 (9.24) | 0.30 |

PSM used 1:1 nearest-neighbour matching without replacement and a 0.2-SD logit caliper. The logistic propensity model included age, parity, symptom duration, IVUS stenosis, ovarian vein diameter, recorded pelvic pooling grade, baseline VAS, and prior DVT. Controls were processed in ascending logit-score order. Continuous and ordinal variables in panel B are mean (SD); prior DVT is n (%). SMDs use unadjusted pooled within-group SDs. The matched OR uses 13 discordant pairs favouring received OVE and 5 favouring IVS alone, with Wald inference on the log OR. Residual imbalance remained.
