# Supplementary material for: Iliac vein stenting with or without ovarian vein embolization for May-Thurner syndrome-associated pelvic venous disorder in women: A received-treatment retrospective cohort study
Source: J Vasc Surg Venous Lymphat Disord. 2026 Oct 14;15(1):102641. doi: 10.1016/j.jvsv.2026.102641 (PMC13769316; doi:10.1016/j.jvsv.2026.102641)
Supplement: Supplementary file 2 [file mmc2.docx]

# Supplementary Table S2. SVP classification distribution

Patient-level SVP categories were assigned from recorded symptom domains, variceal manifestations, and pathophysiologic components available in the individual-patient dataset.

| SVP domain | IVS alone (n=71) | Simultaneous IVS+OVE (n=54) | Staged IVS+OVE (n=59) | P value |
| --- | --- | --- | --- | --- |
| Symptoms domain |  |  |  |  |
| S2 | 36 (50.7) | 19 (35.2) | 17 (28.8) | P=0.030 |
| S3 | 35 (49.3) | 35 (64.8) | 42 (71.2) |  |
| Varices domain |  |  |  |  |
| V0 | 5 (7.0) | 0 (0.0) | 1 (1.7) | P<0.001 |
| V1 | 13 (18.3) | 0 (0.0) | 1 (1.7) |  |
| V2 | 27 (38.0) | 37 (68.5) | 37 (62.7) |  |
| V4 | 26 (36.6) | 17 (31.5) | 20 (33.9) |  |
| Pathophysiology domain |  |  |  |  |
| Obst,BR,Pelv | 0 (0.0) | 29 (53.7) | 19 (32.2) | P<0.001 |
| Obst,LR,Pelv | 13 (18.3) | 13 (24.1) | 19 (32.2) |  |
| Obst,LR | 25 (35.2) | 2 (3.7) | 7 (11.9) |  |
| Obst | 25 (35.2) | 0 (0.0) | 1 (1.7) |  |
| Obst,Pelv | 8 (11.3) | 8 (14.8) | 8 (13.6) |  |
| Obst,BR | 0 (0.0) | 2 (3.7) | 5 (8.5) |  |

SVP = Symptoms-Varices-Pathophysiology; IVS = iliac vein stenting; OVE = ovarian vein embolisation. P values were calculated using chi-square tests across the three received-treatment groups.

Pathophysiology-domain abbreviations: Obst = iliac venous obstruction; LR = left-sided renal/gonadal reflux pattern when recorded; BR = bilateral reflux pattern when recorded; Pelv = pelvic venous reflux/pooling component.
