# Supplementary material for: Iliac vein stenting with or without ovarian vein embolization for May-Thurner syndrome-associated pelvic venous disorder in women: A received-treatment retrospective cohort study
Source: J Vasc Surg Venous Lymphat Disord. 2026 Oct 14;15(1):102641. doi: 10.1016/j.jvsv.2026.102641 (PMC13769316; doi:10.1016/j.jvsv.2026.102641)
Supplement: Supplementary file 3 [file mmc3.docx]

Supplementary Table S3. Sensitivity analyses, extended follow-up, and additional secondary results

**A. Effect estimates**

| **Analysis** | **Estimate** | **95% CI** | **P value** | **Notes** |
| --- | --- | --- | --- | --- |
| Stabilised IPTW, received OVE vs IVS alone | OR 3.12 | 1.09-6.87 | — | Eight-variable propensity model; 99th-percentile truncation; 2000 patient-bootstrap resamples |
| Overlap-weighted marginal analysis | OR 2.42 | 0.66-8.84 | 0.181 | Maximum absolute SMD 0.12 after weighting |
| Overlap-weighted risk difference | 21.2 percentage points | -2.7 to 39.4 |  | Bootstrap CI; 1000 resamples |
| AIPW risk difference | 19.1 percentage points | -9.5 to 39.4 |  | Bootstrap CI; 1000 resamples |
| Simultaneous vs staged OVE | OR 1.31 | 0.52-3.29 | 0.566 | Exploratory; not an equivalence analysis |
| Planned staged vs post-IVS reassessment | OR 2.06 | Not estimated | 0.322 | Fisher exact; 33/45 vs 8/14 |
| OVE received by actual 12-month assessment | aOR 2.44 | 1.13-5.25 | 0.023 | One OVE occurred after assessment |
| Time-updated OVE exposure, repeated VAS | -0.63 VAS points | -0.91 to -0.36 | <0.001 | Gaussian GEE; AR(1); descriptive |

B. Expanded 15-variable propensity-score balance diagnostics (distinct from the eight-variable PSM/IPTW model in Supplementary Table S1)

| **Covariate** | **Before weighting SMD** | **After IPTW SMD** | **After overlap weighting SMD** |
| --- | --- | --- | --- |
| Age_years | -0.01 | 0.04 | 0.01 |
| BMI_kg_m2 | -0.24 | -0.09 | -0.01 |
| Parity | 0.50 | 0.23 | 0.04 |
| Symptom_duration_months | 0.07 | 0.02 | 0.01 |
| IVUS_stenosis_percent | 0.09 | 0.09 | 0.01 |
| Ovarian_vein_diameter_mm | 1.13 | 0.27 | 0.12 |
| Pelvic_venous_pool_grade | 1.33 | 0.67 | 0.11 |
| VAS_Baseline | 0.41 | 0.23 | 0.04 |
| VEINES_QOL_Baseline | -0.35 | -0.05 | -0.00 |
| SF36_PCS_Baseline | 0.02 | -0.10 | 0.01 |
| SF36_MCS_Baseline | -0.00 | 0.06 | 0.00 |
| Prior_DVT | -0.04 | 0.14 | -0.00 |
| Dyspareunia | 0.30 | 0.24 | 0.04 |
| Dysuria_urgency | 0.32 | 0.13 | 0.03 |
| Lower_limb_varicosities | -0.08 | -0.11 | 0.02 |

C. Staged OVE subtype sensitivity

| **Staged subtype** | **n** | **Responders** | **Response rate** | **Mean IVS-to-OVE interval, days** |
| --- | --- | --- | --- | --- |
| planned | 45 | 33 | 73.3% | 174.4 |
| reassessment | 14 | 8 | 57.1% | 157.3 |

**D. Extended available-case outcomes**

| **Month** | **Group** | **Available/due** | **Completeness, %** | **VAS, mean (SD)** | **Response, n/N (%)** | **Stent patency, n/N (%)** |
| --- | --- | --- | --- | --- | --- | --- |
| 24 | Overall | 140/153 | 91.5 | 2.9 (2.3) | 85/140 (60.7) | 139/140 (99.3) |
| 24 | A | 56/61 | 91.8 | 3.2 (2.2) | 30/56 (53.6) | 56/56 (100.0) |
| 24 | B | 41/44 | 93.2 | 2.6 (2.1) | 27/41 (65.9) | 41/41 (100.0) |
| 24 | C | 43/48 | 89.6 | 2.8 (2.5) | 28/43 (65.1) | 42/43 (97.7) |
| 36 | Overall | 98/105 | 93.3 | 2.8 (2.2) | 59/98 (60.2) | 97/98 (99.0) |
| 36 | A | 40/43 | 93.0 | 3.0 (2.1) | 19/40 (47.5) | 40/40 (100.0) |
| 36 | B | 27/28 | 96.4 | 2.9 (2.5) | 16/27 (59.3) | 27/27 (100.0) |
| 36 | C | 31/34 | 91.2 | 2.5 (1.9) | 24/31 (77.4) | 30/31 (96.8) |
| 48 | Overall | 74/80 | 92.5 | 2.7 (2.1) | 48/74 (64.9) | 73/74 (98.6) |
| 48 | A | 29/31 | 93.5 | 2.8 (1.9) | 18/29 (62.1) | 29/29 (100.0) |
| 48 | B | 20/21 | 95.2 | 3.0 (2.5) | 11/20 (55.0) | 20/20 (100.0) |
| 48 | C | 25/28 | 89.3 | 2.4 (2.1) | 19/25 (76.0) | 24/25 (96.0) |
| 60 | Overall | 29/35 | 82.9 | 3.3 (2.8) | 16/29 (55.2) | 28/29 (96.6) |
| 60 | A | 9/12 | 75.0 | 2.8 (2.7) | 5/9 (55.6) | 9/9 (100.0) |
| 60 | B | 12/13 | 92.3 | 3.7 (2.8) | 6/12 (50.0) | 12/12 (100.0) |
| 60 | C | 8/10 | 80.0 | 3.2 (3.3) | 5/8 (62.5) | 7/8 (87.5) |

Due denominators exclude patients whose index date had not yet reached the target month by August 31, 2026. Response was at least 50% VAS reduction from baseline. No observations were carried forward.

**E. Cumulative events through last available follow-up**

| **Group** | **N** | **Follow-up, median (IQR), mo** | **Maximum, mo** | **Lost** | **Not patent** | **Restenosis** | **Thrombosis** | **Recurrence** | **Reintervention** | **Death** |
| --- | --- | --- | --- | --- | --- | --- | --- | --- | --- | --- |
| Overall | 184 | 37.3 (23.7-54.2) | 66.5 | 16 | 4 | 3 | 1 | 27 | 24 | 0 |
| A | 71 | 39.0 (24.6-54.4) | 66.0 | 6 | 0 | 0 | 0 | 11 | 10 | 0 |
| B | 54 | 35.0 (23.6-55.0) | 66.4 | 4 | 1 | 0 | 1 | 6 | 6 | 0 |
| C | 59 | 37.9 (22.2-51.6) | 66.5 | 6 | 3 | 3 | 0 | 10 | 8 | 0 |

F. Secondary outcomes through 12 months

| **Outcome / Timepoint** | **IVS alone (n=71)** | **Simultaneous IVS+OVE (n=54)** | **Staged IVS+OVE (n=59)** | **P value*** |
| --- | --- | --- | --- | --- |
| VAS pain score, median (IQR) |  |  |  |  |
| Baseline | 6.0 (4.9–7.3) | 6.9 (5.6–8.0) | 7.2 (6.0–9.0) | 0.008 |
| 1 month | 4.0 (2.6–5.1) | 4.0 (3.0–4.8) | 5.0 (4.1–5.9) | <0.001 |
| 3 months | 2.7 (1.4–3.8) | 2.7 (2.0–3.6) | 3.1 (2.4–4.2) | 0.065 |
| 6 months | 2.4 (1.1–4.3) | 1.9 (1.0–2.6) | 2.3 (0.9–3.4) | 0.187 |
| 12 months | 2.0 (1.0–4.9) | 1.6 (1.2–3.2) | 1.8 (0.9–4.2) | 0.602 |
| VEINES-QOL, mean (SD) |  |  |  |  |
| Baseline | 48.8 (9.0) | 45.4 (9.3) | 45.7 (10.1) | 0.077 |
| 3 months | 55.5 (9.6) | 59.4 (8.6) | 56.0 (8.5) | 0.041 |
| 6 months | 59.5 (8.3) | 64.8 (8.0) | 61.3 (9.1) | 0.003 |
| 12 months | 60.7 (8.7) | 67.6 (7.7) | 65.1 (7.7) | <0.001 |
| SF-36 PCS, mean (SD) |  |  |  |  |
| Baseline | 40.8 (9.3) | 39.8 (8.5) | 42.0 (8.3) | 0.407 |
| 3 months | 46.4 (8.6) | 49.2 (8.0) | 47.3 (8.1) | 0.169 |
| 12 months | 50.5 (8.1) | 54.6 (7.6) | 52.0 (8.1) | 0.018 |
| SF-36 MCS, mean (SD) |  |  |  |  |
| Baseline | 43.7 (9.2) | 42.8 (9.1) | 44.5 (9.7) | 0.625 |
| 3 months | 48.3 (8.9) | 50.7 (8.4) | 49.4 (8.8) | 0.316 |
| 12 months | 51.1 (9.0) | 53.8 (8.0) | 52.4 (7.7) | 0.199 |
|  | | | | |

**G. Patency, cumulative events, and procedural safety**

| **Outcome** | **IVS alone (n=71)** | **Simultaneous IVS+OVE (n=54)** | **Staged IVS+OVE (n=59)** |
| --- | --- | --- | --- |
| Primary patency at 12 months | 71/71 (100.0) | 54/54 (100.0) | 58/59 (98.3) |
| Restenosis by exact 12-month assessment date | 0 | 0 | 1 |
| Thrombosis by exact 12-month assessment date | 0 | 0 | 0 |
| Not patent at last imaging | 0 | 1 | 3 |
| Restenosis through last imaging | 0 | 0 | 3 |
| Thrombosis through last imaging | 0 | 1 | 0 |
| Symptom recurrence through last contact | 11 | 6 | 10 |
| Any reintervention through last contact | 10 | 6 | 8 |
| Any recorded 30-day event | 19 (26.8) | 18 (33.3) | 16 (27.1) |
| Grade III or higher 30-day event | 0 | 1 (1.9) | 0 |

Exact event dates superseded the original undated binary restenosis and thrombosis fields. Recurrences and reinterventions recorded through extended follow-up all occurred within 12 months.

H. Exploratory subgroup analysis

| **Subgroup** | **IVS alone responders** | **Received OVE responders** | **OR (95% CI)** | **P interaction** |
| --- | --- | --- | --- | --- |
| IVUS stenosis ≤70% | 20/38 (52.6%) | 48/63 (76.2%) | 2.82 (1.21-6.61) | 0.272 |
| IVUS stenosis >70% | 19/33 (57.6%) | 33/50 (66.0%) | 1.42 (0.58-3.47) |  |
| Ovarian vein ≤6 mm | 19/33 (57.6%) | 12/17 (70.6%) | 1.69 (0.50-5.68) | 0.726 |
| Ovarian vein >6 mm | 20/38 (52.6%) | 69/96 (71.9%) | 2.28 (1.06-4.92) |  |
| Symptom duration ≤2 years | 23/39 (59.0%) | 33/49 (67.3%) | 1.43 (0.60-3.38) | 0.248 |
| Symptom duration >2 years | 16/32 (50.0%) | 48/64 (75.0%) | 2.94 (1.22-7.10) |  |
| Prior DVT: No | 32/60 (53.3%) | 70/97 (72.2%) | 2.25 (1.15-4.39) | 0.510 |
| Prior DVT: Yes | 7/11 (63.6%) | 11/16 (68.8%) | 1.25 (0.27-5.90) |  |
|  | | | | |

I. Exploratory multivariable predictor model

| **Variable** | **Unadjusted OR (95% CI)** | **P value** | **Adjusted OR (95% CI)*** | **P value** |
| --- | --- | --- | --- | --- |
| Received OVE (vs IVS alone) | 2.08 (1.12-3.87) | 0.021 | 2.46 (1.08-5.61) | 0.032 |
| Ovarian vein diameter, per mm increase | 1.03 (0.95-1.13) | 0.469 | 0.99 (0.89-1.09) | 0.788 |
| IVUS stenosis >70% (vs ≤70%) | 0.81 (0.44-1.50) | 0.508 | 0.80 (0.42-1.50) | 0.478 |
| Pelvic venous pool grade ≥2 (vs <2) | 1.32 (0.71-2.46) | 0.386 | 0.79 (0.36-1.75) | 0.565 |
| Symptom duration >2 years (vs ≤2 years) | 1.14 (0.62-2.10) | 0.666 | 1.10 (0.58-2.07) | 0.769 |
| Age, per year increase | 1.00 (0.98-1.02) | 0.976 | 1.00 (0.98-1.03) | 0.847 |
| Parity ≥2 (vs <2) | 0.89 (0.48-1.64) | 0.705 | 0.85 (0.45-1.59) | 0.609 |
| Prior DVT (vs no prior DVT) | 1.08 (0.45-2.56) | 0.864 | 1.09 (0.45-2.64) | 0.850 |
|  | | | | |

AIPW = augmented inverse probability weighting; CI = confidence interval; GEE = generalised estimating equation; IPTW = inverse probability of treatment weighting; IVS = iliac vein stenting; OVE = ovarian vein embolisation; SMD = standardised mean difference; VAS = visual analogue scale. Sensitivity, timing, subgroup, and extended follow-up analyses were exploratory.

Panel F notes. VAS is median (IQR), with three-group Kruskal-Wallis P values. Quality-of-life scores are mean (sample SD), with one-way ANOVA P values. All P values are unadjusted.
