# Supplementary material for: Iliac vein stenting with or without ovarian vein embolization for May-Thurner syndrome-associated pelvic venous disorder in women: A received-treatment retrospective cohort study
Source: J Vasc Surg Venous Lymphat Disord. 2026 Oct 14;15(1):102641. doi: 10.1016/j.jvsv.2026.102641 (PMC13769316; doi:10.1016/j.jvsv.2026.102641)
Supplement: Supplementary file 4 [file mmc4.docx]

# Supplementary Table S4. Thirty-day procedural event details

Values are n/N (%). Group C represents staged IVS then OVE. Events were graded using the Clavien-Dindo framework. CTPA = computed tomography pulmonary angiography; IJV = internal jugular vein; LOV = left ovarian vein.

A. Event categories

| Event category | IVS alone | Simultaneous IVS+OVE | Staged IVS+OVE |
| --- | --- | --- | --- |
| Puncture-site ecchymosis | 12/71 (16.9) | 9/54 (16.7) | 9/59 (15.3) |
| Transient chest tightness, CTPA negative | 7/71 (9.9) | 5/54 (9.3) | 5/59 (8.5) |
| Low-grade post-embolisation fever | 0/71 (0.0) | 3/54 (5.6) | 2/59 (3.4) |
| Coil migration requiring snare retrieval | 0/71 (0.0) | 1/54 (1.9) | 0/59 (0.0) |

B. Severity

| Severity | IVS alone | Simultaneous IVS+OVE | Staged IVS+OVE |
| --- | --- | --- | --- |
| Grade I | 19/71 (26.8) | 17/54 (31.5) | 16/59 (27.1) |
| Grade IIIa | 0/71 (0.0) | 1/54 (1.9) | 0/59 (0.0) |

All Grade I events resolved with observation, local compression, oxygen/CTPA rule-out, or physical cooling without antibiotics. The single Grade IIIa event was coil migration from the left ovarian vein to the renal vein ostium; it was retrieved using a snare via IJV access, with one additional hospital day and no sequelae.
